# Different Associations of CD45 Isoforms with STAT3, PKC and ERK Regulate IL-6-Induced Proliferation in Myeloma

**DOI:** 10.1371/journal.pone.0119780

**Published:** 2015-03-17

**Authors:** Xu Zheng, Allison S. Li, Huanyu Zheng, Dongmei Zhao, Dagang Guan, Huawei Zou

**Affiliations:** 1 Department of Oncology, ShengJing Hospital of China Medical University, Shenyang, Liaoning, China; 2 Harvard University, Cambridge, MA, United States of America; 3 He University, Shenyang, Liaoning, China; Imperial College London, UNITED KINGDOM

## Abstract

In response to interleukin 6 (IL-6) stimulation, both CD45RO and CD45RB, but not CD45RA, translocate to lipid rafts. However, the significance of this distinct translocation and the downstream signals in CD45 isoforms-participated IL-6 signal are not well understood. Using sucrose fractionation, we found that phosphorylated signal transducer and activator of transcription (STAT)3 and STAT1 were mainly localized in lipid rafts in response to IL-6 stimulation, despite both STAT3 and STAT1 localizing in raft and non-raft fractions in the presence or absence of IL-6. On the other hand, extracellular signal-regulated kinase (ERK), and phosphorylated ERK were localized in non-raft fractions regardless of the existence of IL-6. The rafts inhibitor significantly impeded the phosphorylation of STAT3 and STAT1 and nuclear translocation, but had little effect on (and only postponing) the phosphorylation of ERK. This data suggests that lipid raft-dependent STAT3 and STAT1 pathways are dominant pathways of IL-6 signal in myeloma cells. Interestingly, the phosphorylation level of STAT3 but not STAT1 in CD45^+^ cells was significantly higher compared to that of CD45^-^ cells, while the phosphorylation level of ERK in CD45^+^ myeloma cells was relatively low. Furthermore, exogenously expressed CD45RO/RB significantly enhanced STAT3, protein kinase C (PKC) and downstream NF-κB activation; however, CD45RA/RB inhibited IL-6-induced ERK phosphorylation. CD45 also enhanced the nuclear localization of STAT3 but not that of STAT1. In response to IL-6 stimulation, CD45RO moved into raft compartments and formed a complex with STAT3 and PKC in raft fraction, while CD45RA remained outside of lipid rafts and formed a complex with ERK in non-raft fraction. This data suggests a different role of CD45 isoforms in IL-6-induced signaling, indicating that while CD45RA/RB seems inhibit the rafts-unrelated ERK pathway, CD45RO/RB may actually work to enhance the rafts-related STAT3 and PKC/NF-κB pathways.

## Introduction

As a growth factor of multiple myeloma (MM) [1–3], interleukin-6 (IL-6) has been presumed to play an essential role in the pathogenesis and proliferation of myeloma cells. Upon binding the IL-6 receptor (IL-6R), IL-6 induces dimerization and subsequent phosphorylation of gp130 with the activation of the Janus kinase (JAK) [4]. Phosphorylated gp130 recruits the signal transducer and activator of transcription (STAT)3, followed by the dimerization, phosphorylation and translocation of STAT3 to the nucleus [5]. In myeloma cells, IL-6 triggers cell proliferation via at least two intracellular signaling pathways, including JAK/STAT3[6] [7] [8], and the ras-dependent mitogen-activated protein kinase (MAPK)/extracellular signal-regulated kinase (ERK) cascade [8–10]. Furthermore, the physiological roles of src family kinase (SFK), Lyn, in myeloma cells have also been examined. The Lyn-specific antisence and the phosphatase inhibitor together obstruct the IL-6-induced proliferation in CD45^+^ myeloma cells, but not in CD45^-^ myeloma cells [8]. Furthermore, Lyn and downstream protein kinase C (PKC) can be activated by IL-6 only in CD45^+^ myeloma cells [11]. This data suggests that except for the activation of STAT and ERK, Lyn/PKC activation via CD45 molecules is also required for IL-6-induced proliferation. Interestingly, down-regulation of Lyn activity does not influence STAT3 and ERK activation in CD45^+^ myeloma cells [8], and the inhibition of ERK activity using ERK inhibitor or siRNA has no effect on the phosphorylation of STAT3 in INA-6 myeloma cells [12], suggesting that there are no cross talk among these three pathways. However, the precise mechanisms underlying the different IL-6 signaling regulation remain poorly understood.

Myeloma cells possess heterogeneous characteristics. This fact was proven by the discovery that 1.) in response to IL-6 stimulation, immature but not mature myeloma cells proliferate markedly [13]; 2.) CD45 is expressed in immature myeloma cells but not in mature cells [14,15] and 3.) Only CD45^+^, but not CD45^-^ myeloma cells, proliferate after IL-6 stimulation [8,16,17]. Considerable evidence indicate that expression of CD45 is essential for the activation of both T cells and B cells [18–20], highlighting the importance of CD45 in regulating immune function not only on T cells and B cells but also on myeloma cells.

CD45 activity and regulation is mainly determined by its localization relative to its substrates, such as SFK [20]. Lipid rafts are specified membrane microdomains in the plasma membrane enriched in cholesterol and sphingolipids [21,22]. These microdomains usually function as triggers to bring different signaling molecules into proximity with their substrates and potentiate the downstream signaling [23]. Strong evidence for lipid rafts-dependent platform of signaling complexes has come from studies on immunoreceptor signaling including BCR, TCR and cytokines [24–26]. Some signaling molecules, such as IL-6R, gp130 and Lyn, are localized in the raft fraction before and/or after cytokine stimulation [17,27], while other molecules, such as CD45, depart from rafts after CD3 stimulation in T cells [28] or move into raft fractions upon BCR engagement in B cells [29] and IL-6 stimulation in myeloma cells [17], suggesting the important regulative role of lipid raft in signaling transduction.

The different isoforms of CD45 are generated by alternative splicing of three exons 4, 5, and 6 [18], and the different isoforms have been found to vary in their ability to modulate TCR signaling and TCR recognition [30,31]. For example, compared to CD45RA, CD45RO is more likely to associate with CD4, CD8 and be more efficient in promoting T cell activation [31–34]. In response to CD3 stimulation, PKC activity increases in CD45RO^+^ cells more so than in CD45RA^+^ cells [35], which themselves have a different ability to proliferate and secrete IL-4 and IFNγ [36]. In myeloma cells, we have previously reported that following IL-6 stimulation, both CD45RO and CD45RB, but not CD45RA, translocated to lipid rafts from outside of raft compartments, where they formed a complex with Lyn and activated Lyn by dephosphorylating Lyn Tyr 507 at the carboxy terminal and phosphorylating Lyn Tyr 396 [17]. This process suggests a positive regulator of CD45RO/RB in IL-6 signaling. Such discriminate associations also suggest that extracellular domains of CD45 isoforms may differentially promote association of the intracellular molecules with specific ability to regulate IL-6 signaling in myeloma. Therefore, additional clarification of the functional difference of CD45 isoforms in myeloma should be another key area of focus.

Here we demonstrate that CD45 isoforms partitioning into raft or nonraft microdomains dictate whether CD45 isoforms function as a positive or negative regulator of IL-6 signal transduction. We show that CD45RO/RB positively regulate STAT3 and PKC/ NF-κB signaling after they move into rafts and recruit with STAT3 and PKC in the rafts compartment. These events require the integrity of membrane rafts, indicating that these membrane microdomains provide a platform for the initiation of CD45RO/RB-mediated IL-6 signaling. On the contrast, CD45RA was recovered only in nonraft soluble fractions and formed a complex with ERK, inhibiting ERK activation only from the nonraft compartment following IL-6 stimulation. Thus, this study provides important insights into CD45 isoforms’ signaling in multiple myeloma cells and suggests a different immunomodulatory role for CD45 isoforms in regulating IL-6-mediated proliferation.

## Materials and Methods

### Cell culture

Myeloma cell lines CD45^+^ U266 and CD45^-^ U266 were separated using a cell sorter (Immunotech Coulter, Hialeah, FL) [8]. CD45^+^ U266 and CD45^+^ ILKM2 were cultured in the presence of IL-6 (2 ng/ml) and conducted IL-6 starvation for 12 hours before IL-6 stimulation. All of the cell lines including CD45^+^ U266, CD45^+^ ILKM2, CD45^-^ U266, CD45^-^ NOP2, and stable transfectant-expressed enhanced green fluorescent protein (EGFP) fusions were cultured in RPMI 1640 medium containing 10% fetal calf serum (FCS; M. A. Bioproducts, Walkersville, MD) [8].

### Flow cytometry

For primary cell analysis, bone marrow mononuclear cells isolated from myeloma patients were obtained with informed consent approved by the Institute Review Board of Shengjing Hospital at China Medical University. Cells were stained with fluorescein isothiocyanate (FITC)-conjugated CD138 (MI 15) and PE-cy5-conjugated CD45 (HI 30). CD45^+^ CD138^+^ and CD45^-^ CD138^+^ primary cells were isolated by a cell sorter. To examine the CD45 isoforms’ expression, cell lines and primary cells were collected, stained with phycoerythrin (PE)-conjugated antibodies including human immunoglobulin G (IgG; 679.1 Mc7), CD45 RO (UCLH1), CD45RB (MT4 [6B6] or CD45RA (2H4) (BD Biosciences Pharmingen, San Diego, CA) and analyzed by a cell sorter.

### DNA constructs and retrovirus-medicated gene transfer

STAT1 and STAT3 cDNA were generated by PCR from CD45^+^ U266 cells. After DNA sequencing confirmation, the STAT1 fragment was digested with *Nhe I* and *BglII*, and the STAT3 fragment was digested with *Nhe I* and Sal*I*. The STAT1 or STAT3 fragment was then subcloned into pEGFP-N1 (Clontech, Palo Alto, CA) to yield STAT1 or STAT3-EGFP, respectively. Both EGFP fusions were then excised with *Nhe I* and *NotI*, respectively, and cloned into the retroviral vector pMXpuro (Clontech). Retrovirus-mediated gene transfer was performed [37], and CD45RO, RB or RA PCR products were cloned into the pEGFP-N1 vector and retroviral pQCXIP vector (Clontech) to generate EGFP fusions as previously reported [17].

### Biological isolation of lipid rafts by sucrose density gradient centrifugation

Preparation of lipid rafts was performed according to the method described previously [17]. Briefly, CD45^+^ U266 cells were either stimulated or non-stimulated with IL-6 (10 ng/ml). Cells were washed twice with ice-cold PBS and lysed with 1 ml lysis buffer containing 0.5% Triton X-100 on ice for 30 minutes. After homogenization, the lysates were combined with an equal volume of 85% sucrose in lysis buffer for a final 42.5% solution. The preparation of discontinuous density gradients was completed with 2.7 ml of 35% sucrose followed by a 1.6 ml layer of 5% sucrose in buffer. Samples were centrifuged in a Beckman TY 80Ti rotor at 55 000 rpm (Beckman Coulter, Hialeah, FL). 12 fractions were collected from the top of the gradient and subjected to Western blot-analysis.

### Nuclear, cytoplasmic protein extraction, immunoblotting, immunoprecipitation, and dot-blotting

Nuclear and cytoplasmic protein extracts were isolated as previously described [38]. For immunoblotting, the cells were lysed in lysis buffer [8,27] and the protein concentration in the lysates was determined by a spectrophotometer. Equal amounts of the lysates were subjected to sodium dodecyl sulfate (SDS) sample buffer and the immunoblotting was performed with primary antibodies as indicated in the figures. Proteins were visualized by chemiluminescence after treatment with secondary antibodies conjugated to horseradish peroxidase. The signal densities of some phosphorylated proteins were normalized to that of the corresponding unphosphorylated proteins. For immunoprecipitation, the cell lysates were first pre-cleared by using protein G agarose beads (Santa Crus Biotech, Santa Cruz, CA) for 2 hours on ice. The protein samples were then incubated with 10 μg of specific antibody for 2 hours, followed by incubation overnight with protein G beads at 4°C. The bead-antibody-protein complex was boiled with reducing buffer for 5 minutes before being transferred into gel lanes for SDS-PAGE. Antibodies were obtained as follows: STAT1, STAT3, ERK1/2, IκB-α, phosphorylated STAT1 (P-STAT1, Tyr 701), phosphorylated STAT3 (P-STAT3, Tyr 705), phosphorylated ERK1/2 (P-ERK, Thr202/Thr204), phosphorylated IκB-α (P-IκB, Ser32) and phosphorylated PKC (P-PKC, Ser660) from Cell Signaling Technology. CD45 (35-Z6), SH2 containing tyrosine phosphatase (SHP-2) (B-1), son of sevenless homolog 1 (SOS1) (C-23), CD71 (H-300), PKC-β, SP-1 (1C6), gp130 (C-20) and IL-6R (H-300) were purchased from Santa Cruz Biotech. Flotillin-2 was purchased from BD Biosciences Pharmingen. Dot-blotting was used to identify the lipid rafts’ fractions. Horseradish peroxidase-conjugated cholera toxin B (CTB; List Biology Laboratory, Campbell, CA) was used to label endogenous GM1 ganglioside.

### Proliferation assay

CD45^+^ U266 cells and CD45^+^ CD138^+^ primary cells were cultured in 96-well plates for 72 hours with or without IL-6 (10 ng/ml). PKC inhibitor Ro31-8220 (1 μM), or NF-κB inhibitor BAY11-7082 (5 μM) was obtained from Sigma Chemical Co. and added 1 hour prior to IL-6 stimulation. 5-bromo-2’-deoxyuridine (BrdU) was used to examine the cell proliferation by incorporating BrdU into cellular DNA using a peroxidase-labeled anti-BrdU antibody.

### Live fluorescence imaging

Stable transfectants of STAT1-EGFP and STAT3-EGFP were plated on a 35 mm poly-L-lysine-coated glass-bottom culture dish (Matsunami glass, Tokyo, Japan). Fluorescence-expressing living cells were maintained at 37°C and images were acquired with a laser confocal microscope (LAM 510; Carl Zeiss, Jena, Germany). In order to destroy membrane lipid rafts, a lipid rafts inhibitor methy-β-cyclodextrin (MCD; sigma Aldrich, St Louis, MO) was used. Cells were pretreated with 10 mM MCD for 30 minutes at 37°C followed by IL-6 stimulation.

### Statistical analysis

Data analysis was performed using one-way analysis of variance (ANOVA) and the post-hoc multiple comparisons were performed by using the Tukey honestly significant difference (HSD) test. Results are presented as a mean ± SD of at least three independent experiments, and statistical significance was calculated as indicated.

## Results

### Lipid rafts are necessary for IL-6-induced STAT3 and STAT1 phosphorylation, but not essential for ERK

**Fig 1:**
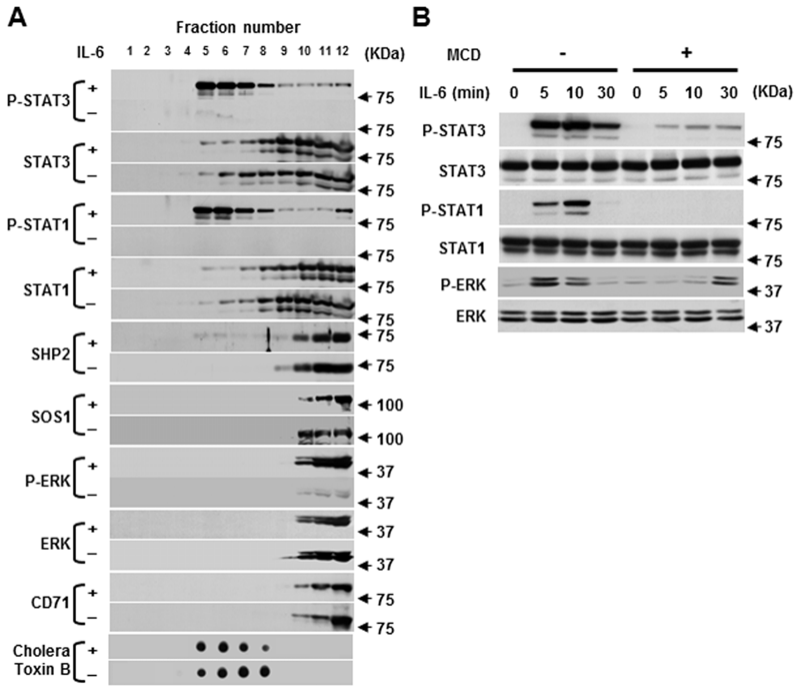
IL-6-induced STAT3 and STAT1 phosphorylation is required for the integrity of lipid rafts. (A) CD45^+^ U266 cells were grown in IL-6 free medium for 12 hours (IL-6 starvation). Cells were incubated with or without 10 ng/ml of IL-6 for 5 minutes. The cell lysates were subjected to sucrose density gradient centrifugation, and endogenous proteins indicated beside figures from each sucrose fraction were analyzed by immunoblotting. CD71 was detected as a nonraft marker. Lipid raft fractions were confirmed using CTX dot plots for each fraction. (B) CD45^+^ U266 cells were treated or untreated with MCD (10 mM) for 30 minutes at 37°C. Cells were then stimulated with IL-6 at different time points. Whole-cell lysates were subjected to SDS/PAGE and separate plots with antibodies are shown. The representative blots of three independent experiments are shown.

First, we examined whether IL-6 signaling molecules are distributed in lipid rafts of myeloma cells. To prepare lipid rafts, CD45^+^ U266 cell lysates were subjected to a sucrose gradient ultracentrifuge and the gradient samples were fractionated into 12 fractions. As shown in Fig. 1A, both STAT3 and STAT1 were detected in both the raft and soluble fractions regardless of the presence of IL-6. IL-6 treatment induced the phosphorylation of both STAT3 and STAT1 and movement of P-STAT3 and P-STAT1 into the rafts fractions. Surprisingly, SHP2, SOS1 and ERK were not significantly distributed in the lipid raft fraction. Although IL-6 could induce tyrosine phosphorylation of ERK (P-ERK), P-ERK did not exist in the raft fraction. Raft marker CTB, which binds GM1 gangliosides, was detected only in the raft fraction (around fraction 5–8), while CD71, a nonraft marker [17,29], was found in virtually every soluble fraction (fractions 10–12). To determine the importance of P-STAT3 and P-STAT1 translocation to lipid rafts in intracellular signaling events, the activation of these molecules was monitored in CD45^+^ U266 cells depleted of cholesterol using MCD. IL-6 treatment resulted in time-dependent STAT1 and STAT3 phosphorylation. Such a response was dramatically abrogated in MCD treatment cells compared to untreated control cells (Fig. 1B). On the other hand, MCD could not effectively block IL-6-medicated activation of ERK. It seems that MCD abrogates the early stage of the activation of ERK but not the late stage. Overall, this data suggests that IL-6-mediated activation of STAT3 and STAT1, but not ERK, is specifically required for the integrity of lipid rafts.

### Lipid rafts are necessary for IL-6-induced STAT3 and STAT1 nuclear localization

**Fig 2:**
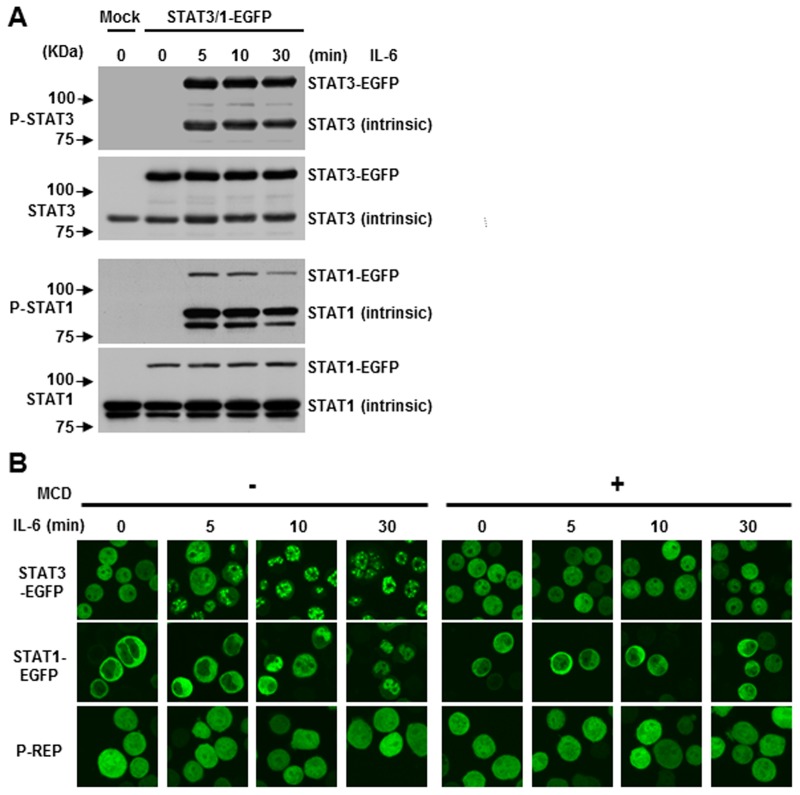
IL-6-induced S.TAT3 and STAT1 nuclear translocation is required for the integrity of lipid rafts. (A) CD45^+^ U266 cells were transfected with untagged (mock) or STAT1-EGFP, STAT3-EGFP expression plasmids, and either treated or untreated with IL-6 at different time points. Immunoblotting was performed as above. (B) Subcellular distribution of STATs-EGFP fusion proteins. Nuclear translocation of both STAT3-EGFP and STAT1-EGFP was evaluated by live cell imaging. Cells were pre-incubated with or without 10 mM MCD for 30 minutes and then stimulated with 10 ng/ml of IL-6 and images were generated at different time points. P-REP was used as a mock vector, which expresses EGFP. Data shown are representative of three experiments.

It is well known that in response to stimulation by IL-6, tyrosine phosphorylated STATs undergo dimerization and are translocated from cytoplasm to nucleus [39,40]. To evaluate STAT’s nuclear trafficking in a live cell, stable transfectants of STAT1-EGFP and STAT3-EGFP in CD45^+^ U266 cells were stimulated with IL-6. Then, specific tyrosine 701 for STAT1 and 705 for STAT3 phosphorylation were examined. Both EGFP-tagged and untagged STAT1 and STAT3 exhibited robust tyrosine phosphorylation with IL-6 stimulation (Fig. 2A), suggesting the potential biological function of STAT3-EGFP and STAT1-EGFP. To further confirm whether IL-6-induced activation of STATs is dependent upon lipid rafts, we carried out real-time microscopic imaging of the translocation of STATs to the nucleus. As shown in Fig. 2B, stably expressed STAT3-EGFP in CD45^+^ U266 cells was localized in the cytoplasm and nucleus even after IL-6 removal for 12 hours. STAT3-EGFP was found to be prominently nuclear soon after the IL-6 stimulation. The peak nuclear fluorescence intensity was about 10 minutes. However, STAT1-EGFP was clearly mainly localized in the cytoplasm prior to treatment with IL-6, which was different from STAT3-EGFP. The fluorescence intensity of the nucleus only rapidly increased between 10 and 30 minutes and peaked at 30 minutes. Furthermore, when STAT3 and STAT1-EGFP expressed cells were treated with MCD, a cholesterol sequestrating agent, the IL-6-induced STAT1 and STAT3 nuclear translocation was blocked. Our previous report already found that MCD could significantly inhibit IL-6-induced proliferation in CD45^+^ U266 cells [17]. Therefore, current data suggests that the integrity of lipid rafts is also required for nuclear localization of STATs and may potentiate subsequent proliferation in multiple myeloma cells.

### Different regulation of CD45 on IL-6-induced protein phosphorylation and nuclear translocation

**Fig 3:**
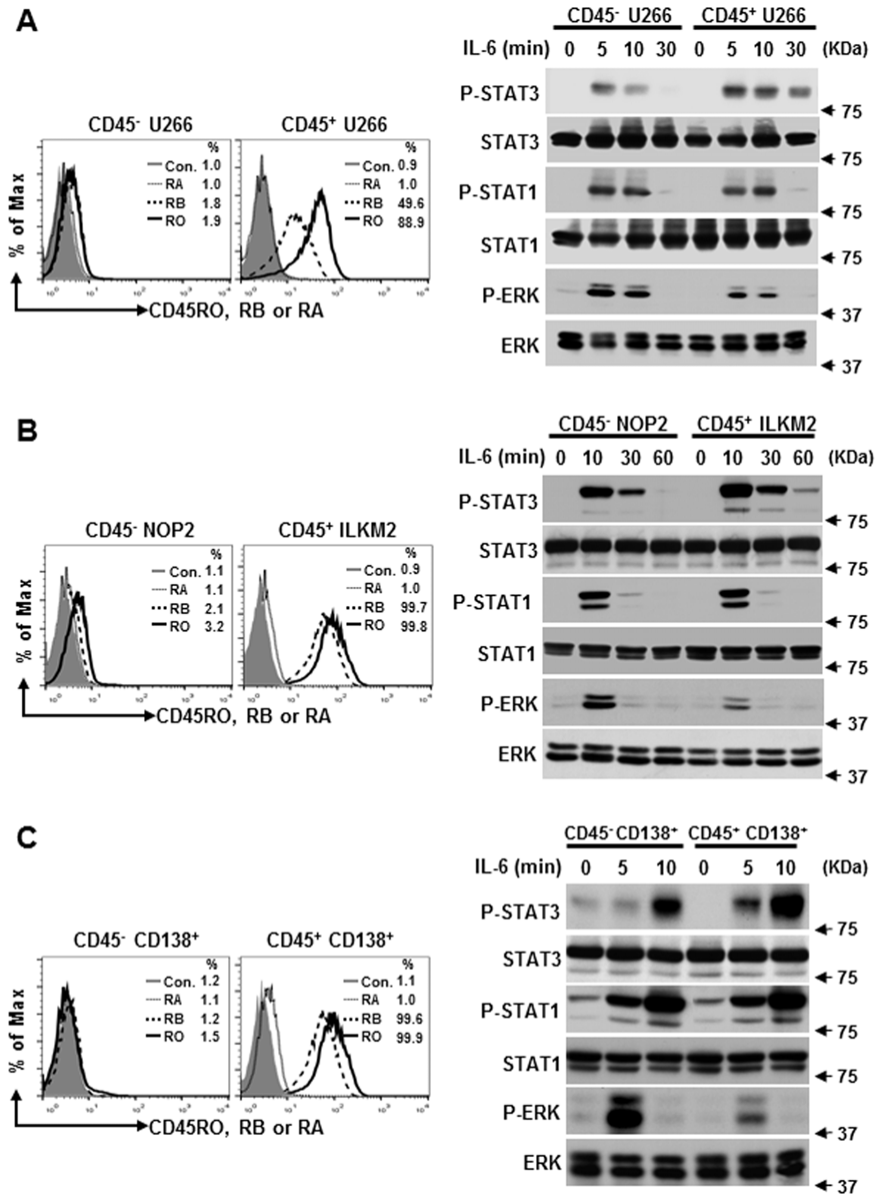
Phosphorylation levels of STAT3 and ERK are different between CD45^+^ and CD45^-^myeloma cells. CD45^-^ U266 cells and CD45^+^ U266 cells (A), CD45^-^ NOP2 cells and CD45^+^ ILKM2 cells (B), and CD45^-^ CD138^+^ and CD45^+^ CD138^+^ primary cells (C) were stained for CD45RO, RB and RA antibodies and analyzed by flow cytometry. The percentage expression relative to its isotype control is shown in the histogram. Cells were also stimulated with IL-6 for the indicated time. Western blotting was performed by using specific antibodies and the representative blots of three independent experiments are shown.

We have demonstrated that CD45 RO, RB, but not RA move to lipid rafts and facilitate IL-6-induced signaling [17]. This study was then aimed at understanding the mechanism behind IL-6-induced proliferation regulated by different CD45 isoforms. First, we carefully evaluate whether the phosphorylation level of IL-6-mediated signaling molecules differed between CD45^+^ and CD45^-^ myeloma cells. As shown in Fig. 3A, IL-6 stimulation induced specific phosphorylation of STAT1, STAT3 and ERK. Surprisingly, the tyrosine phosphorylation level of STAT3, but not STAT1, was greatly enhanced in CD45^+^ U266 cells, which are CD45RO^+^, CD45RB^+^, CD45RA^-^, in comparison with the cells without expression of CD45. In contrast, ERK phosphorylation was significantly reduced in CD45^+^ U266 cells. Because IL-6 elicits distinct responses and activates various signaling pathways in a cell-specific manner [8,37], we evaluate our results in other cell types. Similar to CD45^+^ U266, ILKM2 myeloma cells were also highly expressed by CD45RO, RB but not for CD45RA expression (Fig. 3B). Western blotting data clearly showed that IL-6-induced STAT3 phosphorylation level was markedly higher in CD45^+^ ILKM2 cells, while ERK phosphorylation level was significantly lower. To rule out the cell line based phenomena, primary patient samples were also included and similar results were obtained by comparing CD45^+^ CD138^+^ with CD45^-^ CD138^+^ myeloma cells (Fig. 3C). This data suggests that CD45 (CD45RO, CD45RB) may upregulate IL-6-induced STAT3 tyrosine phosphorylation while downregulating ERK tyrosine phosphorylation.

**Fig 4:**
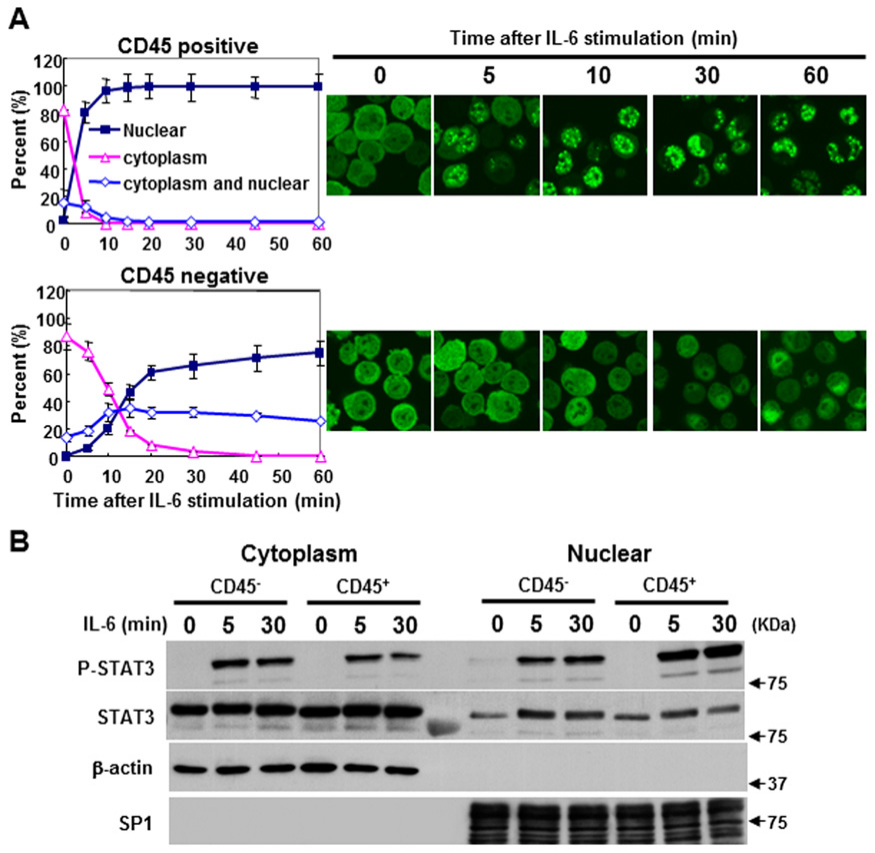
CD45 expression enhances the nuclear localization of STAT3. (A) CD45^-^ U266 and CD45^+^ U266 cells transfected with STAT3-EGFP were analyzed by confocal microscopy for EGFP fusion protein. After stimulation with 10 ng/ml of IL-6, the distributions of STAT3-EGFP fusion protein in nuclear, cytoplasm, or cytoplasm plus nuclear were quantified by counting 100 cells at different time points. The mean percentages from three independent experiments were calculated. (B) Cytoplasm and nuclear extracts were isolated from CD45^-^ U266 cells and CD45^+^ U266 cells, respectively, and analyzed by western blotting with antibodies against phosphorylated STAT3 or STAT3. Cytoplasmic marker β-action and nuclear marker SP1 were used to indicate the purity of our extraction procedure. Data shown are representative of three experiments.

We further investigate whether nuclear localization of STAT3 is different between CD45^+^ and CD45^-^ myeloma cells. Here, using stable transfectants of STAT3-EGFP, we found that IL-6 stimulation induced a markedly quicker and stronger nuclear translocation in CD45^+^ U266 cells compared to that of CD45^-^ U266 cells (Fig. 4A). However, no significant difference was found in STAT1-EGFP nuclear localization between CD45^+^ and CD45^-^ U266 cells (data not shown). We further confirmed this finding using the biochemical method. With this purpose, cytoplasmic and nuclear extraction from both CD45^-^ and CD45^+^ U266 cells were prepared. As shown in Fig. 4B, IL-6 stimulation induced significant phosphorylation of STAT3 both in CD45^-^ and CD45^+^ U266 cells. Importantly, as a consequence of IL-6 stimulation, a significant proportion of phosphorylated STAT3 was translocated into the nuclear compartment isolated from CD45^+^ cells, which was mirrored by a decrease in the cytoplasm compartment. The phosphorylation level of STAT3 induced by IL-6 markedly increased in nuclear extract isolated from CD45^+^ U266 compared to extract isolated form CD45^-^ U266 cells. Simultaneously, the phosphorylation level of STAT3 clearly decreased in the cytoplasmic extract purified from CD45^+^ U266 cells. This data indicates that CD45 (CD45RO, CD45RB) may facilitate IL-6-induced nuclear translocation.

### Raft-targeted CD45RO/RB enhances STAT3 activation and STAT3/CD45RO complex formation

**Fig 5:**
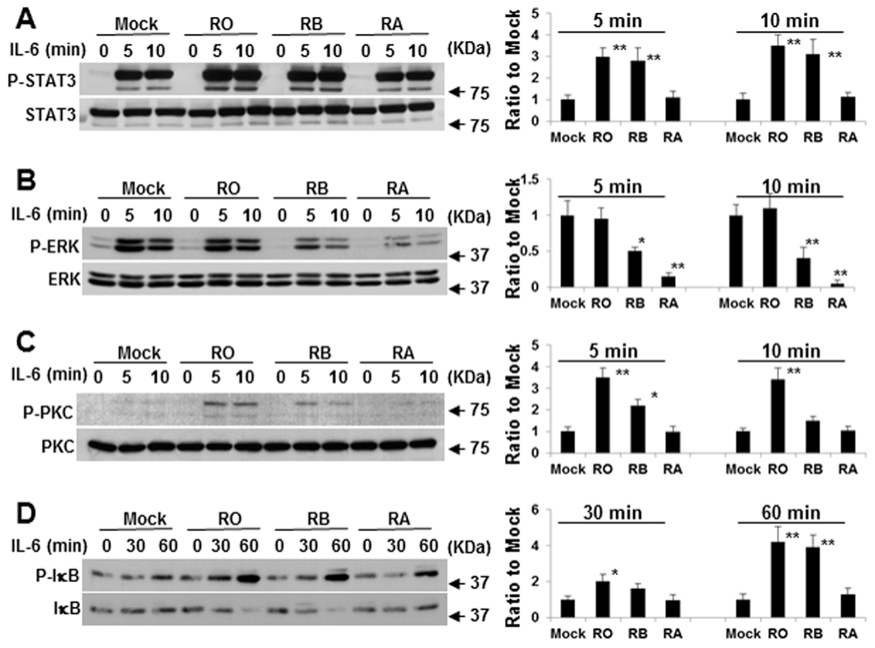
Effects of Exogenously expressed CD45RO, RB, or RA-EGFP on phosphorylation of STAT3, MAPK, PKC and IκB. CD45^-^ U266 cells were transfected with untagged (mock) or CD45RO-EGFP, CD45RB-EGFP or CD45RA-EGFP expression plasmids, and either treated or untreated with IL-6 (10 ng/ml) at different time points. The representative blots are from three independent experiments and separate blotting using antibodies to P-STAT3, STAT3 (A), P-ERK, ERK (B), P-PKC, PKC (C) and P-IκB, IκB (D) are shown. The densities of protein bands were determined by densitometry and the data represent a change from the control mock density. * *p* < 0.05 vs mock control in the presence of IL-6; ** *p* < 0.01 vs mock control in the presence of IL-6 by a one-way ANOVA with HSD test.

**Fig 6:**
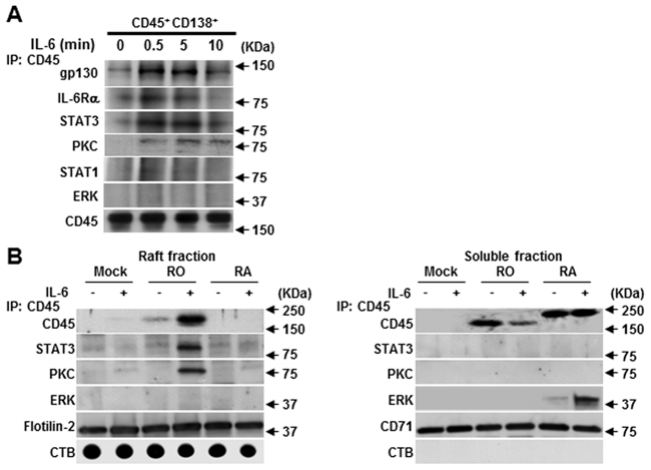
CD45RO forms a complex with STAT3, PKC in lipid rafts, while CD45RA forms a complex with ERK in soluble fraction. (A) CD45^+^ CD138^+^ primary myeloma cells were stimulated with IL-6 (10 ng/ml) for the indicated time, immunoprecipitated (IP) with anti-CD45 antibody, and put through western blotting by using specific antibodies. (B) CD45^-^ U266 cells transfected with untagged (mock), CD45RO-EGFP, CD45RB-EGFP or CD45RA-EGFP expression plasmids were incubated with or without 10 ng/ml of IL-6 for 5 minutes. Lysates were prepared and separated by sucrose fractionation. Raft fraction (5–8) and soluble fraction (10–12) were mixed, respectively, and immunoprecipitated with anti-CD45 antibody followed by western blotting. Raft-targeted protein flotilin-2, CTB and raft-untargeted protein CD71 were performed for quality control.

The effect of CD45RO/RB overexpression on IL-6-induced STAT3 phosphorylation was analyzed to address the question of whether the enhancement of STAT3 phosphorylation is relevant to CD45RO/RB. CD45RO-EGFP, CD45RB-EGFP or CD45RA-EGFP was transfected into CD45^-^ U266 cells to produce CD45RO^+^, CD45RB^+^ or CD45RA^+^ U266 cells, respectively. Overexpression of CD45RO or CD45RB significantly enhanced IL-6-induced STAT3 phosphorylation (Fig. 5A). In contrast, CD45RA did not affect IL-6-induced STAT3 phosphorylation. We next examined whether CD45RO associates with STAT3 in lipid rafts in response to IL-6 stimulation. Using primary CD45^+^ CD138^+^ cells, we first demonstrated that CD45 forms a complex with the IL-6R and gp130. Interestingly, IL-6 also triggered CD45 to form a complex with STAT3, but not STAT1 and ERK (Fig. 6A). Anti-CD45 antibody immunoprecipitation followed by immunoblotting confirmed that CD45RO moved into raft fractions in response to IL-6 stimulation. Importantly, CD45RO moved into raft fractions and coprecipitated with STAT3 but not in soluble fraction (Fig. 6B). This data indicates that when stimulated with IL-6, CD45RO translocates to the lipid raft, forms a complex with IL-6R complex and STAT3, and further potentiates IL-6-induced STAT3 activation.

### Nonraft-targeted CD45RA forms a complex with ERK and antagonizes IL-6-induced ERK phosphorylation

We next determined the reason why the ERK phosphorylation level was lower in CD45^+^ myeloma cells in comparison to CD45^-^ myeloma cells (Fig. 3). As described above, CD45RB^+^ or CD45RA^+^ U266 stable transfectants were prepared. Overexpression of CD45RB or CD45RA substantially inhibited IL-6-induced ERK phosphorylation at multiple time points (Fig. 5B). Conversely, ERK phosphorylation was not affected by CD45RO overexpression. Interestingly, we succeeded in detecting ERK coprecipitation with CD45RA in soluble fraction, but not in raft fraction in response to IL-6 stimulation (Fig. 6B). These results imply that CD45RA may associate with ERK and function as a phosphatase to dephosphorylate ERK outside of lipid rafts.

### CD45RO/RB, but not RA, activates PKC and NF-κB which is necessary for IL-6-induced proliferation

NF-κB induces cell proliferation by regulating the expression of cyclin D and E [41,42]. In B cells, upon antigen ligation, PLC-γ2 is activated, followed by the activation of PKC-β and IKK α/β. Nuclear translocation of active NF-κB contributes to the phosphorylation of IκB [43]. We have found that the PLC-γ2 complex with Lyn and PKC can only be activated in CD45^+^ U266 cells [11]. In this study, exogenously expressed CD45 RO or RB was cloned into CD45^-^ U266 cells to assess their involvement in IL-6-induced PKC, NF-κB activation. As shown in Fig. 5C and 5D, overexpression of CD45RO and CD45RB significantly enhanced the phosphorylation of PKC and NF-κB while the phosphorylation levels of PKC and NF-κB in CD45RA^+^ U266 cells resembled the mock control U266 cells. The lipid rafts translocation of phosphorylated PKC in response to IL-6 stimulation has been demonstrated using a discontinuous sucrose gradient and the requirement of lipid rafts intact in IL-6-induced PKC activation was also confirmed by using raft inhibitor (data not shown). Importantly, IL-6 induced the complex formation of CD45RO with PKC especially in the raft fraction but not in the soluble fraction (Fig. 6B). Thus, this data indicates that CD45RO forms a complex with PKC in raft compartments which may facilitate increased phosphorylation of PKC.

**Fig 7:**
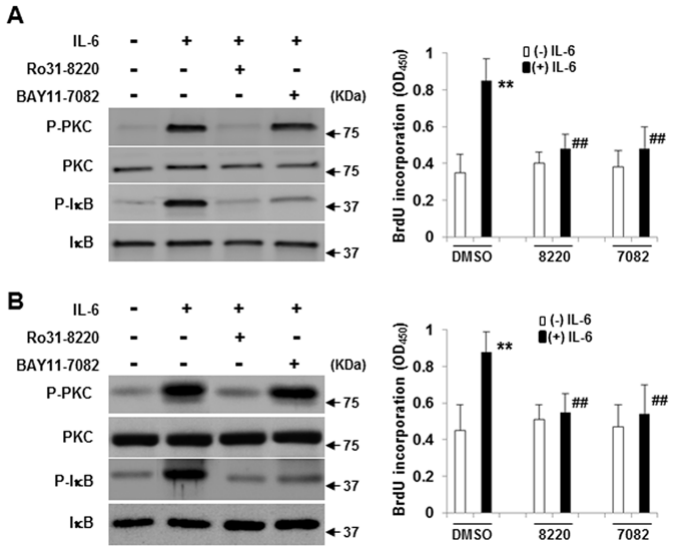
PKC and downstream NF-κB are required for IL-6-induced proliferation in CD45^+^ myeloma cells. CD45^+^ U266 cells (A) or CD45^+^ CD138^+^ primary cells (B) were incubated with or without PKC inhibitor Ro31-8220 (1 μM), or NF-κB inhibitor BAY11-7082 (5 μM) for 1 hour and stimulated with IL-6 (10 ng/ml). PKC phosphorylation level was measured after 10 minutes of stimulation with IL-6, and the IκB phosphorylation level was analyzed after 60 minutes stimulation. Western blotting was performed by using specific antibodies. The representative blots of three independent experiments are shown. BrdU incorporation was used to detect the DNA synthesis by IL-6 after 72 hours. DMSO is used as a control. Data are shown as mean ± SD of triplicate cultures and are from one experiment representative of three performed. ** *p* < 0.01 vs DMSO control in the absence of IL-6; ## *p* < 0.01 vs DMSO control in the presence of IL-6 by a one-way ANOVA with HSD test.

We further used reagents that specifically inhibit PKC and IκB to evaluate whether PKC, NF-κB activation were involved in IL-6-induced proliferation. As shown in Fig. 7, treatment of CD45^+^ U266 cells (Fig. 7A) or CD45^+^ CD138^+^ cells (Fig. 7B) with PKC inhibitor abrogated IL-6-induced PKC and IκB phosphorylation, while the NF-κB inhibitor only affected IκB phosphorylation instead of PKC phosphorylation. Furthermore, both inhibitors triggered similar effects on IL-6-induced proliferation confirmed by both the myeloma cell line and primary cells. These results suggest that the activation of PKC and its downstream molecule NF-κB are especially required for IL-6-induced proliferation.

## Discussion

Although CD45 plays an important role in regulating the IL-6-induced proliferation, the question of the downstream signaling difference of CD45 isoforms in myeloma cells remains poorly documented. This study provides evidence that IL-6-induced STATs (but not ERK) phosphorylation and nuclear translocation are required for the integrity of lipid rafts. Importantly, stimulation of myeloma cells with IL-6 increased the proportion of CD45RO in the raft fractions, where CD45RO formed a complex with IL-6R, gp130, STAT3 and PKC, that could potentiate the activation of STAT3, PKC and downstream NF-κB. However, CD45RA remained outside of the rafts compartment, formed a complex with ERK, and inhibited the IL-6-induced ERK phosphorylation.

Lipid rafts have been regarded as a signaling platform on the cell membrane where different signal pathways can be initiated [21,44]. IL-6 signaling molecules, such as IL-6R, gp130 and Lyn, have been detected in the raft fractions before and after IL-6 stimulation [17]. Here, our sucrose gradient centrifugation analysis revealed that IL-6-mediated activation of STAT3 and STAT1 occurs in the lipid rafts fraction, whereas IL-6-mediated activation of ERK can only take place in nonlipid raft regions. The importance of lipid rafts in IL-6-induced STATs activation was also confirmed by using a lipid raft inhibitor, as both STAT3 and STAT1 phosphorylation was impaired by the cholesterol-depleting drug MCD. It is apparent that cholesterol is essential for IL-6-induced STATs activation because MCD also disrupts the nuclear translocation of STATs. The pivotal role of cholesterol in IL-6-induced STATs activation and myeloma cells proliferation [17] suggests that administration of cholesterol-depleting drugs could be a potential therapeutic strategy against IL-6-induced myeloma cell proliferation. Our previous data already demonstrated that recruitment of the IL-6R complex to lipid rafts and CD45 activates Lyn by inducing a Lyn membrane confirmation change [17]. It is also reported that SFK, which is enriched in lipid rafts, mediates integrin-induced tyrosine phosphorylation by providing a docking site for adapter proteins Grb2 and Shc [45]. This may be similarly applied to receptor tyrosine kinases that require their residency in lipid rafts and docking site occupation to activate STAT3 and STAT1. Lipid rafts have been identified to be a novel and effective therapeutic target in cancer [46]. Specially, phospholipid ether edelfosine accumulates in myeloma cell membrane rafts and promotes apoptosis of cancer cells through co-clustering of Fas/CD95 death receptor and lipid rafts [47]. STAT3 has also been considered to be a target for the induction of apoptosis in tumor cells [48] and Gossypol, a cotton plants extraction, has been found to induce apoptosis in myeloma cells by inhibiting phosphorylation of JAK and STAT3 [49]. Therefore, our data suggests that a combination of rafts target drug with JAK/STAT3 inhibitor seems to be a very promising approach for myeloma therapeutic intervention.

Our findings establish a relationship between CD45 isoforms’ microdomain locations and the regulation of IL-6-mediated myeloma cell activation. Here, we show that IL-6-induced STAT3 tyrosine phosphorylation is significantly enhanced in CD45^+^ myeloma cells in comparison with CD45^-^ myeloma cells. Consistent with the enhanced phosphorylation of STAT3 is the observation that STAT3 nuclear translocation is quicker and more efficient in CD45^+^ STAT3—EGFP transfectants cells. Phosphorylation and nuclear localization of STAT1 in response to IL-6 is comparable in CD45^+^ and CD45^-^ myeloma cells, indicating that CD45 does not regulate STAT1 phosphorylation. An interesting finding is that CD45RO/RB, but not CD45RA, enhances the phosphorylation of STAT3-induced by IL-6, followed by the formation of the CD45RO:STAT3 complex in raft fractions, but not in nonraft ones. It is not clear why this difference was observed. However, the specific raft movement of CD45RO/RB but not CD45RA [17], as well as the rafts location of phosphorylated STAT3, is consistent with this finding. Our data showing that CD45RO forms a complex with STAT3 is supported by evidence that CD45 associates with JAK and directly regulates tyrosine phosphorylation of STAT3 and STAT5 in interferon-α (IFN-α)-induced signaling in COS cells [50]. The increased STAT3 phosphorylation in CD45RO/RB overexpression cells further indicates that CD45 isoforms’ expression patterns affect IL-6-induced myeloma cell proliferation possibly through interactions with different CD45 isoforms, because CD45RO has been indicated to homodimerize more efficiently [51] and more readily with the immunological synapse [52] than with other highly molecular weight isoforms. These studies suggest a rapid, ligand-dependent interaction of CD45 with IL-6R complex and the physical association of CD45RO/RB with STAT3 may be potent ways of increasing the ability of IL-6-induced myeloma cells to proliferate. It should be noted that our results are consistent with a previous report in which CD45 is positive regulator for IFN-α-induced JAK-STAT signaling in Jurkat cells [53]. Our data now demonstrates that it is raft-associated CD45-RO/RB, but not raft-excluded CD45RA, that functions as a positive regulator of STAT3, but not STAT1, and processive IL-6 signaling transduction. Compared to CD45^-^ myeloma cells, CD45^+^ myeloma cells with an activated JAK/STAT3 pathway are particularly sensitive to JAK2 inhibitor [54] which inhibits IL-6-induced JAK and STAT3 phosphorylation. On the other hand, CD45RO, but not CD45RA and pan CD45 antibody, significantly inhibits proliferation and STAT3 phosphorylation triggered by IL-2 and IL-4 in human lymphoblasts, indicating the functional differences between CD45 isoforms [55]. Our findings are of clinical interest because they predict multiple target drugs for myeloma, such as raft target drug, JAK/STAT inhibitor and CD45RO antibody, and thus may be more efficacious and less vulnerable to acquire resistance.

We propose that lipid rafts are involved in early ERK activation, because abolition of lipid rafts with MCD results in the attenuation of the early ERK waves of activation. The small amount of ERK upstream molecule, SHP2, in the raft fraction can also explain this phenomenon. In contrast, the late activation remains constant in cells pretreated with MCD, suggesting that the late signaling response of ERK leading to phosphorylation is indicative of another originating source that is not in lipid raft domains. Because the CD45RA:ERK complex formation is induced by IL-6 in nonraft compartments, we favor a model in which CD45RA dephosphorylation of nonraft ERK is facilitated by different isoforms of Ras activation. An interesting report elucidates different raft compartments and requirements for K-ras and H-ras isoforms, in which the K-ras isoform is known to most efficiently induce Raf-1 (and thus ERK), selectively localizing to nonraft microdomains. Alternatively, the H-ras isoform initially localizes to lipid rafts and then relocates to nonrafts upon GTP binding [56]. It is therefore possible that CD45RA is involved in K-ras-mediated ERK activation outside of lipid rafts. An alternative explanation pertains to potential synergistic cooperation of CD45RA with other membrane β-galactoside-binding lectins, such as galectin-1. Because galectin-1 prefers to bind highly glycosylated CD45RA [20], but not CD45RO, this results in the inhibition of ERK activation [37]. A previous study implicates the interaction of galectin-1 and fibronectin, inhibition of ERK phosphorylation, induction of P21, P27, inhibition of CdK2 activity and ultimately growth inhibition also support this suggestion [57,58]. Finally, it remains to be determined whether nonraft-localized CD45RA inhibits ERK activation due to CD45 activity on other nonraft-localized ERK regulators that we have yet to examine. Together with our findings demonstrating increased CD45RA:ERK association in myeloma cells, these results raise the possibility that CD45RA localized outside of lipid rafts attenuates K-ras-triggered ERK activation by facilitating CD45RA:galectin-1:fibronectin interaction and thus induction of P21, P27 and subsequent inhibition of CdK2 activity and myeloma cells growth inhibition.

Our findings also elucidate a previously unrecognized mechanism by which CD45 isoforms differentially regulate IL-6 signaling in myeloma cells. The expression of raft-targeted CD45RO/RB enhances STAT3 activation, while raft-excluded CD45RA attenuate ERK activation. These findings prompted us to consider alternative mechanisms of STAT3 activation that might be facilitated by raft-targeted CD45RO/RB. We considered the possibility that raft-targeted CD45RO/RB induces the activation of PKC/NF-κB pathways directly, obviating an absolute requirement for Lyn phosphorylation for PKC activation. Indeed, IL-6-induced dephosphorylation at position Tyr507 and phosphorylation at position Tyr396 occurs simultaneously with the translocation of CD45 RO to lipid rafts [17]. Our previous report also shows that inhibition of Lyn activity significantly blocks the proliferation-induced by IL-6 in CD45^+^ myeloma cells, but does not affect either STAT3 or ERK activation [8]. Specially, other groups demonstrate the inhibition of ERK without interference with phosphorylation of STAT3 [12], as well as the inhibition of arsenic trioxide on IL-6-induced STAT3 but not ERK activation in myeloma cells [59]. Our findings that overexpression of CD45RO enhances CD45RO:PKC complex formation, phosphorylation of PKC and NF-κB, as well as the proliferation inhibition by both PKC and NF-κB inhibitors, suggest the functional and biological significance of CD45RO in IL-6-inducd independent Lyn/PKC/NF-κB activation.

**Fig 8:**
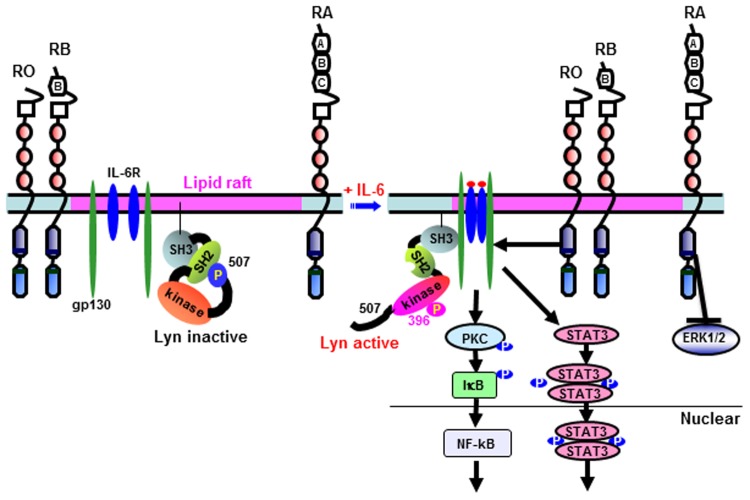
Model of CD45 isoforms-mediated IL-6 signaling in multiple myeloma cells. Engagement of IL-6R with IL-6 leads to complex formation of IL-6R, gp130, Lyn as well as CD45RO/RB in raft microdomains. In response to IL-6 stimulation, CD45RO/RB moves into lipid rafts to induce dephosphorylation of the negative regulatory of Tyr507, phosphorylation of Tyr396, and subsequent conformation change and Lyn activation [17]. We confirmed our hypothesis that lipid rafts-targeted CD45RO/RB facilitates IL-6-induced STAT3 and Lyn/PKC/NF-κB activation in rafts microdomains, while raft-excluded CD45RA remains outside of lipid rafts after IL-6 stimulation and negatively regulates ERK-activation.

In conclusion, we propose a model of CD45 isoforms-mediated IL-6-induced myeloma cells signaling (Fig. 8). The data presented here extends the finding from our previous research that demonstrates the important regulative role of CD45 isoforms in IL-6-induced myeloma cells proliferation [17]. Using our previous report in conjunction with our current findings, we propose that membrane rafts provide a platform for CD45RO/RB recruitment to STAT3, Lyn and PKC, which functions as a positive regulator to enhance IL-6-induced STAT3, NF-kB signaling. However, CD45RA, which stays in the non-rafts fraction regardless of the IL-6 stimulation, likely acts as a phosphatase to inhibit ERK activation following engagement of IL-6 stimulation.
